# Five years’ experience with value-based quality improvement teams: the key factors to a successful implementation in hospital care

**DOI:** 10.1186/s12913-022-08563-5

**Published:** 2022-10-20

**Authors:** Kirsten Daniels, Marc B. V. Rouppe van der Voort, Douwe H. Biesma, Paul B. van der Nat

**Affiliations:** 1 Department of Value-Based Healthcare, St. Antonius Hospital, P.O. Box 2500, 3430 EM, Nieuwegein/Utrecht, the Netherlands; 2 Radboud University Medical Center, Radboud Institute for Health Sciences, Scientific Center for Quality of Healthcare (IQ healthcare), Nijmegen, the Netherlands; 3 Department of Health Organisation, Policy and Economics, Faculty of Health Sciences, Maastricht University, Maastricht, the Netherlands; 4 Department of Internal Medicine, Leiden University Medical Center, Leiden, the Netherlands; 5 Department of Internal Medicine, University Medical Centre Utrecht, Utrecht, the Netherlands

**Keywords:** Value-based healthcare, VBHC, Quality improvement, Health outcomes, Shared-decision making, Lean

## Abstract

**Background:**

In recent years, value-based healthcare (VBHC) has become one of the most accepted concepts for fixing the ‘broken’ healthcare systems. Numerous hospitals have embraced VBHC and are trying to implement value-based quality improvement (VBQI) into their practice. However, there is a lack of knowledge on how to practically implement VBHC and organizations differ in their approach. The aim of this study was to explore the main factors that were experienced as hindering and/or supporting in the implementation of VBQI teams in hospital care.

**Methods:**

A qualitative study was performed with semi-structured interviews with 43 members of eight VBQI teams in a large Dutch top-clinical teaching hospital. Participants included physicians, physician assistants, nurses, VBHC project leaders, managers, social workers, researchers and paramedics. Interview grids were structured according to the RE-AIM model (reach, effectiveness, adoption, implementation and maintenance). A thematic content analysis with open coding was used to identify emerging (sub)themes.

**Results:**

We identified nine main factors divided over three domains (organization, culture and practice) that determined whether the implementation of VBQI teams was successful or not: 1). Practical organization of value-based quality improvement teams, 2). Organizational structure 3). Integration of VBHC with existing quality improvement approaches and research 4). Adoption and knowledge of the VBHC concept in the hospital 5). Multidisciplinary engagement 6). Medical leadership 7). Goal setting and selecting quality improvement initiatives 8). Long-cycle benchmarking and short-cycle feedback 9). Availability of outcome data.

**Conclusions:**

Overall, this study goes beyond the general VBHC theory and provides healthcare providers with more detailed knowledge on how to practically implement value-based quality improvement in a hospital care setting. Factors in the ‘organization’ and ‘practice’ domain were mentioned in the strategic value agenda of Porter and Lee. Though, this study provides more practical insight in these two domains. Factors in the ‘culture’ domain were not mentioned in the strategic value agenda and have not yet been thoroughly researched before.

**Supplementary Information:**

The online version contains supplementary material available at 10.1186/s12913-022-08563-5.

## Introduction

In recent years, value-based healthcare (VBHC) has become one of the most accepted concepts for fixing the ‘broken’ healthcare systems around the world. Numerous hospitals have embraced VBHC and are trying to implement value-based quality improvement (VBQI) into their practice. However, there is a lack of knowledge on how to practically implement VBHC and organizations differ in their approach. Therefore, it is important to learn from prior experience and evaluate what elements of these implementation methods work, and which do not. Until now, the implementation of VBHC has been evaluated only to a limited extent. In a large Dutch top-clinical teaching hospital, multidisciplinary value-based quality improvement (VBQI) teams have been implemented for several medical conditions since 2015. The aim of this study was to explore the main factors that were experienced as hindering and/or supporting in the implementation of VBQI teams in hospital care.

In 2006, the concept of VBHC was introduced by Porter and Teisberg [1] in response to the ‘broken’ healthcare system in de United States. Since European healthcare systems struggle with some of the same issues – namely rising healthcare costs and fragmented care delivery - VBHC has also made its way into Europe. The aim of VBHC is to increase patient value, which is defined as the best possible patient-relevant health outcomes and patient experience divided by the costs to achieve those outcomes [1, 2]. To support the value transformation, Porter and Lee [3] introduced the strategic value agenda with six steps: 1. ‘Organize into integrated practice units (IPUs)’, 2. ‘Measure outcomes and costs for every patient’, 3. ‘Move to bundled payments for care cycles’, 4. ‘Integrate care delivery across separate facilities’, 5. ‘Expand excellent services across geography’, and 6. ‘Build an enabling information technology platform’. The value agenda provides first guidance on the implementation of VBHC in a healthcare organization but it is still insufficient with regards to detailed information on how to practically implement VBHC in a healthcare organization.

Over the last years, several healthcare organizations in Europe have been experimenting with the implementation of VBHC [4], each in their own way. Unfortunately, little is known about the factors that made these implementation efforts successful or not. Globally, most emphasis seems to be placed on the first and second step of Porter and Lee’s value agenda: ‘organizing into Integrated Practice Units’ and ‘measuring outcomes and costs for every patient’. Measuring and improving outcomes and communicating about these outcomes to patients is a step that some healthcare organizations already seem to make [5]. However, the step from ‘care as usual’ to organizing into IPUs is big and seems to be an incremental process of organizing care around the patient rather than a radical change [6]. Although some research is done on the clinical effect of specific IPUs [7–9], no research is yet available on the organizational aspects of successfully embedding an IPU in the context of a general hospital. Some studies described the implementation of VBQI teams; a team that is focused on improving the outcomes of one specific disease [5, 10–14]. A qualitative study from Sweden [11] concluded that VBQI participants appreciated VBHC because of the focus on creating patient value instead of minimizing costs, as is common in many other ‘regular’ management concepts. Another qualitative study from Sweden [15] concluded that VBHC implementation requires a continuous learning journey in which it is essential to challenge health professionals’ ideas and beliefs.

To our knowledge, there are no studies available yet that evaluate which factors are important to a successful implementation of VBQI in hospital care. The goal of the present study is to provide healthcare providers with more knowledge on how to practically implement VBQI in a hospital care setting.

## Method

### Design

An exploratory, qualitative design with semi-structured individual interviews was used. The interview guide (Additional file 1: Appendix A) was structured according to the RE-AIM model [16], a frequently used model for the evaluation of a health intervention implementation process. The RE-AIM model consists of five dimensions; reach, effectiveness, adoption, implementation and maintenance. 1). Reach is defined as the size and characteristics of the target population that is subjected to the intervention. 2). Effectiveness refers to the impact of the intervention on potential positive and negative outcomes. 3). Adoption is defined as the number of people involved in the implementation of the intervention. 4). Implementation indicates the degree to which the intervention was successfully implemented into the organization. 5). Maintenance refers to the long-term continuation and institution’s adoption of the intervention.

### Setting

This study took place in a Dutch top-clinical teaching hospital that started implementing VBQI teams in 2014. At the time of the interviews (2019), VBQI teams were implemented for eighteen different medical conditions. The present hospital is a member of a larger network (Santeon) of seven leading teaching hospitals in the Netherlands that jointly work together towards value-based healthcare [5, 14]. In 2020, they received the VBHC Prize for their collaborative work on the ‘Santeon better together’ program [17].

### VBQI teams

Eight VBQI teams were included in this study; breast cancer, lung cancer, prostate cancer, colorectal cancer, hip arthrosis, chronic kidney failure, hip fracture (trauma geriatrics), and the sleep center. The first six teams were selected based on their maturity as improvement team: each of them had completed at least two improvement cycles. Hip fracture and the sleep center were selected based on their experience in organizing care around the patient; i.e. disease-oriented organization. They had an advanced form of multidisciplinary organization and quality improvement and more formal management. The hip fracture and sleep center teams had already organized care around the patient and were setting up a quality improvement cycle similar to the other six participating teams. Including the hip fracture and sleep center teams as well as the other six ‘mature’ provided us with the opportunity to further investigate whether the order of setting up a VBQI team matters or not.

The VBQI teams consist of six to sixteen members, representing the main specialties involved in the care for the specific patient group. The teams consist of physicians, nurses, managers, and medical specialists of supporting departments such as radiologists and hospital pharmacists. Each team is chaired by a “medical leader” (physician), and supported by a value-based healthcare project leader and data analyst from the central support staff (0.2 FTE, VBHC department). A detailed overview of team membership is provided in Additional file 2: Appendix B.

### Value-based improvement cycle

**Fig. 1:**
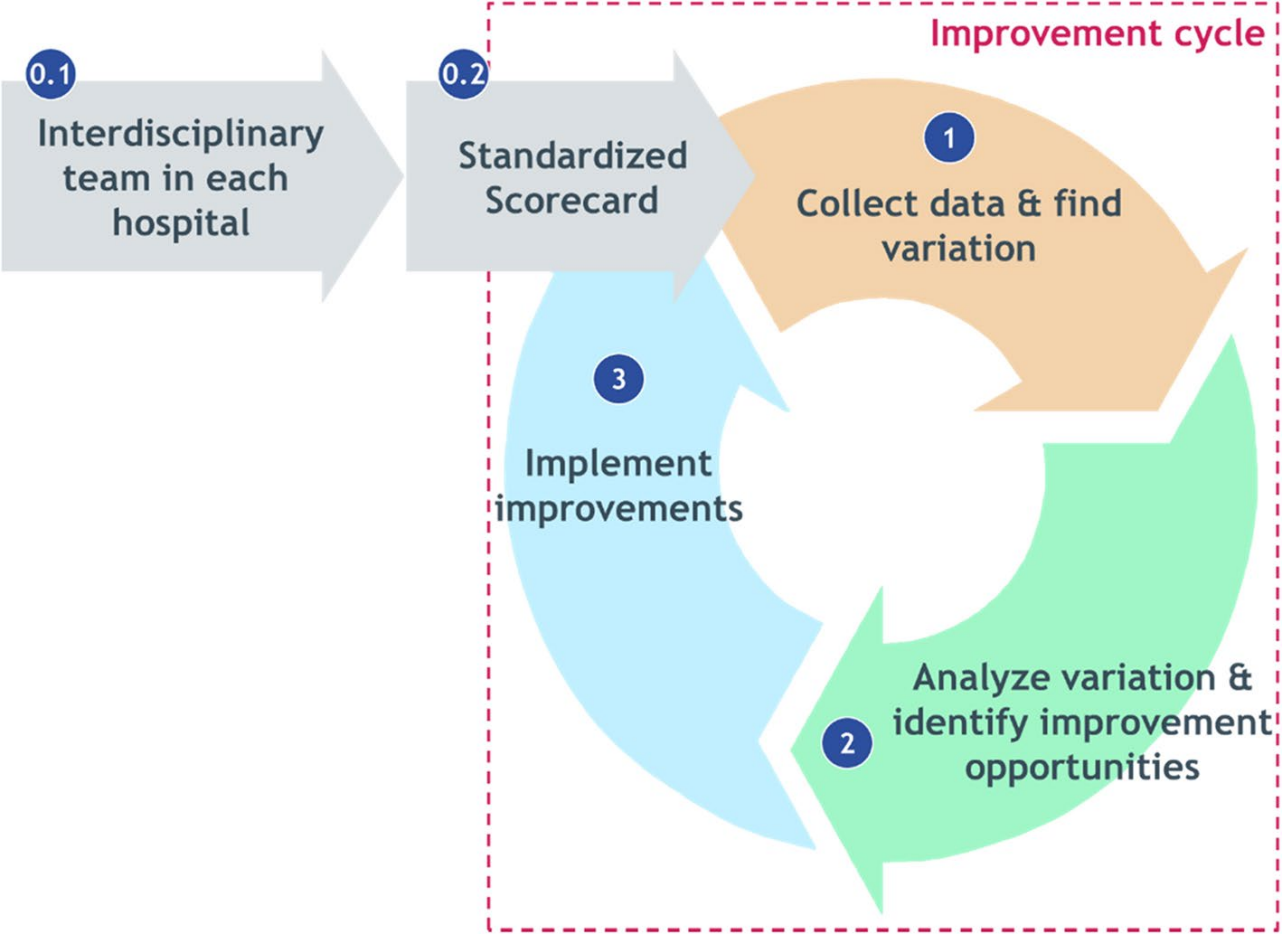
Santeon improvement cycle [18]

Six of the eight participating VBQI teams worked towards value-based care by measuring and benchmarking their treatment outcomes with partnering hospitals. As part of the ‘better together’ program [17], a network of seven top-clinical teaching hospitals in the Netherlands formed a learning platform where treatment outcomes are measured, benchmarked and improved twice a year following the Santeon Improvement Cycle (Fig. 1). The Santeon Improvement Cycle was developed by Santeon and the Boston Consulting Group (BCG) [5, 14]. In the Santeon Improvement Cycle, each improvement team goes through three-to-five stages: Forming a multidisciplinary group of healthcare professionals involved in the care for patients with the medical condition in question (0.1), defining which outcomes and case-mix variables need to be measured (0.2), collecting data and finding variation in outcomes between the seven networking hospitals (1), analyzing the variation in outcomes by looking at potential practice variation between the hospitals and adopt a best practice from one of the hospitals or select an improvement initiative from literature (2), implementing the improvement initiatives (3). When the third stage is completed, the cycle starts over again with collecting data and evaluating the impact of the implemented improvement initiative. In addition to the Santeon improvement cycle with networking hospitals, the VBQI teams in the present hospital also measure and improve their outcomes on their own.

### Data collection

In total, forty-three members of VBQI teams were invited to participate in this study. All members accepted the invitation and participated. Participants were of different professions (Table 1). The average duration of interviews was 72.3 minutes, ranging from 21.1 minutes to 88.1 minutes. All interviews were in Dutch, audio-recorded and transcribed verbatim. Four interns supported the organization, audio-recording, taking notes and transcription of the interviews. During the period in which the interviews took place, two meetings were organized with all interviewers and interns to discuss preliminary results. Data was collected in the period from January 2019 to June 2019.

**Table 1:** Characteristics of participants

	***N*** = 43
Sex, male	25 (58,1%)
*Occupations*	
Physicians	19 (44,2%)
Managers/Department heads	8 (18,6%)
Nurses	6 (14,0%)
VBHC project leaders	5 (11,6%)
Paramedics	2 (4,7%)
Researchers	1 (2,3%)
Physician assistants	1 (2,3%)

### Data analysis

A thematic content analysis was carried out. First, two independent researchers (KD and PBVDN) used open content coding to find factors that participants indicated as important in the implementation of VBQI teams. The two researchers independently coded the transcripts in sets of five and found consensus on the coding after each set. The code book was then adjusted accordingly. They continued until saturation of information was reached, which occurred after coding 20 interviews. The remaining interviews were coded according to the code book established in the first 20 interviews. The analyses were performed with use of ATLAS.ti software (version 8.4.18). For readability, the quotes placed in the results section of this paper were translated from Dutch to English by the main researcher (KD).

## Results

We identified nine themes and several subthemes divided over three domains, that were relevant in the implementation of VBQI in hospital care (Table 2). These factors were sometimes experienced as hindering (−), as supporting (+), or both (+/−), depending on the poor/proper implementation of the factor. The factors displayed in Table 2 were relevant for all participating teams, but whether the experience was positive or negative differed between teams.

**Table 2:** Important factors in the implementation of value-based quality improvement in hospital care

Domain	Main factor	Sub factors	Experience^§^
**Organization of VBQI**	1. Practical organization of value-based quality improvement teams	a. Available time of health professionals	-
		b. Planning and attendance of meetings	-
		c. Availability of VBHC support staff (data analysts/project leaders)	+
	2. Organizational structure	a. Organization of care around the patient	+/-
		b. Volume versus value for patients	-
		c. Shared workspace	+/-
		d. Health professionals dedicated to medical condition	+
		e. Financial benefits of adopting VBHC concept	+
		f. Formal responsibility for quality of care	+/-
		g. Mandate of value-based quality improvement team	+/-
	3. Integration of VBHC with existing QI approaches and research	a. Shared Decision Making	+
		b. Lean-philosophy	+
		c. Use of Patient Reported Outcome Measures (PROMs)	+
		d. Scientific Research	+
		e. one central group for quality improvement	+/-
**Culture of VBQI**	4. Adoption and knowledge of the VBHC concept in the hospital	a. Knowledge of VBHC concept	+/-
		b. Belief in added value VBHC	+/-
		c. Reputation of VBHC concept	+/-
		d. Impact of seeing VBHC results	+/-
	5. Multidisciplinary engagement	a. Engagement of multiple disciplines in improvement team	+/-
		b. Small efficient teams	+
		c. Equal input from team members	+
		d. Outcome data that is relevant and adjustable for all team members	-
		e. Patient involvement	-
		f. Engagement of health professionals outside improvement team	-
		g. Readiness to change	-
		h. Engagement of colleagues from other participating hospitals	+/-
	6. Medical leadership	a. Inspirational medical leadership	+
		b. Medical leader’s ability to engage others	+
		c. Involvement/accessibility of medical leader	+/-
**Practice of VBQI**	7. Goal setting and selecting quality improvement initiatives	a. Purpose of value-based quality improvement team meetings	-
		b. Setting clear goals for outcome improvement	-
		c. Selection of improvement initiative; need for clear methodology	-
		d. Improving without clear improvement potential	-
		e. Improving quality of care without outcome data	-
		f. Evaluation of improvement initiatives	-
	8. Long-cycle benchmarking and short-cycle feedback	a. Long-cycle benchmarking between networking hospitals	+/-
		b. Short-cycle continuous improvement through electronic care pathways	+
	9. Availability of outcome data	a. Data collection and data analysis	-
		b. Access to data not directly related to an intervention	-
		c. (national) Data registry	+
		d. Support of information technology (IT)	+

^§^Factors were experienced by participants as hindering (-), as supporting (+), or both (+/-), depending on the poor/proper implementation of the factor

### Practical organization of value-based quality improvement teams

Participants mentioned that the practical organization of the VBQI teams was challenging at times. Many of the team members are physicians or nurses whose time is limited due to their demanding duties in daily clinical practice (subtheme a). Currently, time spent on VBQI is considered participants’ ‘free time’. Some participants suggested that it would be easier to participate in the VBQI team if this would be ‘scheduled time’.

In addition, participants state that it is often difficult to plan a meeting where all team members are present (subtheme b). Planning seemed to be especially more difficult for larger teams. Whether or not a meeting is scheduled within regular working hours (often meetings are scheduled in the evening), influences the attendance rate during the meeting. In turn, this has consequence for the level of continuity, level of support for QI initiatives and the level of multidisciplinary input; i.e. the extent to which all relevant disciplines provide input. However, due to team members’ other priorities or obligations, the attendance rate is sometimes low.

Participants also mentioned that the availability of VBHC support staff in the practical organization of VBQI was important for a successful implementation (subtheme c). Data analysts and project leaders from the VBHC department support the quality improvement teams through data collection, data analysis, connecting health professionals and structuring the process. In general, participants were positive about the involvement of VBHC support staff. However, some participants mentioned that there is need for more support in the practical daily organization of the improvement teams. Especially in the planning of meetings and gathering of people, medical leaders ask for more support:

*‘I am doing the logistic management [practical daily organization] of the improvement team now, but I am also busy with the preparations, so I am doing those logistic things in addition. Next to all the other hundred things on my plate. So I would really benefit from someone who helps me with that..’*

### Organizational structure

Most participants indicated that it was difficult to move towards care around the patient (subtheme a). Traditionally, hospitals are structured in departments, each with their own expertise (e.g. oncology, surgery, neurology etcetera) and participants indicated that it is difficult to fully shift towards disease-oriented organization. However, participants in the hip fracture and sleep center teams explain that they have incrementally made the move towards disease-oriented organization. According to one of the participants of the sleep center team, the shift towards a disease-oriented organization was made possible because a prior hospital merger served as stimulus to change their way of delivering care:

*“When our hospital took over the other hospital, we also took over the independent sleep center that was part of it. We are now integrating this independent sleep center into the hospital. And we see that the integration motivates us [the sleep center] to change even more [towards disease-oriented organization]. […] I think you need a stimulus in order to change.*

In line with the difficulty to organize care around the patient, participants mention that putting value over volume is still difficult (subtheme b). Hospital care is still payed according to pay-for-volume contracts, and budget responsibility still lies with the traditional functional departments. Participants also claimed that there is need for a shared workspace where all health professionals, who are involved in the care for a specific patient group, work together and meet each other face-to-face (subtheme c). In contrast, the hip fracture and sleep center teams have multidisciplinary consultation hours where they meet, which was valued by the participants. Furthermore, participants preferred health professionals who are dedicated to a specific condition, to ensure engagement in the VBQI team and quality of care (subtheme d):

*‘I think it is important that you specialize in something, also as a physiotherapist or a dietician. […] I think every disease requires a different treatment and if you know more about it you can perform it better. So I think you should be organized around a condition which is better for the health professional as well’.*

Some participants argued that there should be financial benefits for adopting the VBHC concept (subtheme e) to stimulate other specialists to start with VBQI as well. Others suggested the opposite, namely a financial penalty for departments that do not embrace the VBHC concept.

Many participants mentioned that a formal responsibility for their patient group’s quality of care could increase team members’ involvement (subtheme f). Suggestions such as being a ‘self-directed team’ and ‘having our own financial budget’ were made. Though, other participants questioned how much influence a formal responsibility would have on team members’ involvement.

Opinions differed with regards to the VBQI team’s mandate. Some participants stated that they were content with the current mandate to implement changes in the various involved departments (subtheme g). Some specified that the medical leader should have formal mandate to make decisions about the patient group in question together with the team. However, others mentioned that the mandate is always a shared responsibility.

### Integration of VBHC with existing quality improvement approaches and research

A number of other quality improvement approaches were considered to be stimulating factors for the implementation of VBQI within the hospital setting. The reason for this stimulating effect is the overlap in the use of outcome data. Participants mentioned that outcome data collected by the VBQI team can also be used in Shared Decision Making (subtheme a). The same applies to implementing the lean-philosophy (subtheme b): some participants state that efforts to apply the lean-philosophy in a department could be combined with efforts to work towards more value-based care delivery. The use of patient-reported outcome measures (PROMs) was also specifically mentioned as a stimulating factor (subtheme c), because PROMs provide enriched data which supports VBQI on a patient level (patient-physician consults) as well as on population level (benchmarking/continuous improvement).

Apart from several quality improvement approaches that were mentioned, multiple participants mentioned the positive influence of performing scientific research alongside implementing VBQI (subtheme d): outcome data was more easily available to the VBQI team when scientific research was conducted for that particular medical condition.

However, the presence of multiple other QI approaches was sometimes also seen as hindering because of the lack of coordination and joint approach. Participants mentioned that some topics are discussed twice and some topics do not reach the VBQI team because they are being discussed in other meetings. Having various quality improvement approaches/meetings can be confusing and inefficient, as described by two participants:

*‘…improvements are introduced hospital-wide. And that is what makes it so complex. Because healthcare delivery is being improved from several different angles. But there is nobody who oversees all that. Even within the department, it is difficult to inform people about all that has been improved.’*

*‘But you know what is difficult now, there are so many meetings. I go from a meeting with the networking hospitals to a meeting about the electronic care pathway, to a meeting about the national outcome registry, sometimes I really don’t remember which meeting I’m in.’*

It seems that integration of VBHC with other QI initiatives, in the form of better coordination and a joint approach, would lead to synergy. In line with this finding, some participants said it would be beneficial to have one team that addresses all quality-improvement related topics such as PROMs, SDM and the lean-philosophy for a particular medical condition (subtheme e).

### Adoption of the VBHC concept

Participants stated that within the VBQI teams, people were fully educated about the VBHC concept. Yet, some health professionals outside of the VBQI team did not have adequate knowledge on VBHC (subtheme a) nor did they fully believe in the added value of VBHC (subtheme b). Also, the reputation of the VBHC concept within the organization was not always positive (subtheme c). Some participants said that their colleagues outside the improvement team saw VBHC as a yet another new management tool to cut costs.

A supporting factor is ‘seeing the results of VBHC’ (subtheme d). When participants and health professionals outside the improvement teams saw the first results of VBHC, their believe in the added value of VBHC increased. The underlying reason seems to be that seeing the positive impact of an improvement initiative on health outcomes motivated the members to continue their work in the VBQI team. Vice versa, waiting for results was seen as a hindering factor: not being able to see the results of an improvement effort was considered demotivating. One of the participants therefore called for starting with small short-term improvement initiatives rather than big long-term improvement initiatives:

*‘The best way is of course if you can just quickly show that VBQI works “We’ve researched this, we have proposed these changes and this is the outcome. Look, it works! The hospital stay is shorter, or we have fewer complications or lower costs.” Of course, if you can quickly show these results, people tend to believe in it and join in. But if it’s a very long process with a lot of discussion going on, then people do wander off a bit at some point.’*

### Multidisciplinary engagement

Most participants stated that all necessary disciplines were represented in their VBQI team. Though some teams pointed out that multidisciplinary engagement was sometimes challenging, especially when the medical condition in question was very complex and multiple medical supporting units such as pathology and radiology were involved (subtheme a). Furthermore, the engagement of multiple disciplines, especially the engagement of multiple physicians, could make it more difficult to find consensus on which improvement initiatives to select. In line with that, other participants stated that they worked well in smaller, more efficient teams with only the lead health professionals (subtheme b). The other disciplines were consulted separately on emerging issues.

Furthermore, participants claimed that it was meaningful to have equal input from all team members so that all are equally responsible and equally motivated to participate (subtheme c). Especially the input from nurses was found important since they work closely with the patient. Additionally, participants state that in order to involve all disciplines in VBQI, outcome data should be relevant and adjustable for all disciplines (subtheme d), as described by this participant:

*‘You know, it is nonsense, I sit there as a physiotherapist and yes I could give some input. But the discussion is rarely about my medical actions. Same goes for inviting a radiologist, an anesthetist or an OR assistant; they are not going to spend that much time on it if they do not benefit from it.’*

Regarding the involvement of patients in the VBQI team, experiences differed between teams (subtheme e). Some teams stated that they had never involved patients in their VBQI but wanted to. Other teams said that they had involved patients in the past by inviting them to participate in the meetings where outcomes and improvement initiatives were discussed, but felt that the way they had executed it was not very productive. One of the arguments that was made was that patients can often only speak for their own situation, and not for the whole population. One of the participants referred to this issue as the ‘*N* = 1 experience’. Yet, involving a patient in the VBQI meeting could also provide a humane dimension to the discussion. Another participant made the suggestion to involve patients on a consultative basis, such as via a focus group. In general, it seems that there is need for more guidance on how to involve patients in VBQI, as described by one of the participants:

*‘I would very much like to have one person in the quality department who is specialized in patient participation who can tell us how to handle that. And what tools can you use and what you need for patient participation.’*

It was also found essential to engage health professionals outside of the VBQI team (subtheme f) since all health professionals involved in the care for a particular patient group need to support the implementation of improvement initiatives.

Furthermore, participants experienced that it is challenging to make changes in an organization when health professionals are not ready to change (subtheme g). Moreover, the engagement of colleagues from other hospitals was indicated as significant (subtheme h) as discussing outcomes with colleagues increased knowledge on best practices and decreased practice variation. However, some participants also stated that the lack of engagement from other participating hospitals could be demotivating: while some teams were content with the input from other hospitals, others felt that their colleagues from other hospitals did not put in the same amount of effort as they did.

### Medical leadership

According to participants, inspirational medical leadership was an important factor (subtheme a). Inspirational leadership was defined by participants as being decisive, motivated, innovative and respected in the professional field. According to participants, strong leadership was also characterized by the ability to engage others (subtheme b).

*‘You need two things. You need a group of physicians who go for it and who also engage others. Those physicians need to be passionate. But you also really need a leader who pulls through. Arranging something multidisciplinary with each other is very difficult. You need someone with a long breath and pull-through capacity.’*

Furthermore, participants stated that the medical leader needs to be involved and accessible (subtheme c). Some teams claimed to be quite successful due to the active involvement of the inspirational medical leader. However, other teams mentioned that their team was not living up their full potential because the medical leader was busy and not always able attend the scheduled meeting.

### Goal setting and selecting quality improvement initiatives

Participants declared that the purpose of the VBQI team meetings was sometimes unclear to members of the team and healthcare professionals outside the improvement team (subtheme a). Furthermore, when looking at the process of increasing value, participants stated that jointly setting clear goals for each outcome indicator, e.g. raising survival rates by 10%, is an important and often over-looked step in VBQI (subtheme b).

Participants mentioned that there was need for a clearer methodology on how to select improvement initiatives based on improvement potential identified in the data (subtheme c). According to some participants, there was a lot of discussion in the meetings about the outcome data, but it did not always result in the selection of an improvement initiative. Furthermore, it was stated that their hospital’s outcomes often fall within or above the average of benchmarking hospitals or national range of acceptance. There is a lack of knowledge on how to improve care when no clear improvement potential can be found in the data (subtheme d).

*‘I think that it is a disadvantage of this improvement team, that the outcomes of a certain intervention are already so good and generally do not deviate much from the national standards. That the differences are so small, that you are less likely to explain the connection between outcomes and clinical practice.’*

The same problem was mentioned when there was no data available yet (subtheme e). Some participants suggested that it was not possible to perform VBQI, others suggested that it was difficult to start with VBQI without data, but not impossible. Another participant mentioned that there was time and energy spent on investigating the outcome data and implementing the improvement initiative, but that there was little effort spent on the evaluation of the implemented improvement initiative afterwards (subtheme f):

*‘Quite a lot of initiatives have been implemented. Some of them are even pioneering. And after a while, we try to see whether it was implemented properly, but in the meantime we have also already started with a few new projects. As a result, the projects that were previously implemented, are not evaluated properly’.*

### Long-cycle benchmarking and short-cycle feedback

Many participants considered benchmarking of outcome data between the seven participating hospitals motivating (subtheme a). Seeing differences in outcome data and discussing ways of working, protocols and procedures underlying those differences with health professionals from the other participating hospitals was found inspiring and educational. However, some participants also claimed that the benchmarking process sometimes was challenging because it takes a relatively long time to see results.

*‘It is really good that everyone makes their data available. As a result, everyone is really exposed. We also regularly e-mail back and forth between hospitals about “how did you actually arrange this?”. The fact that we are really open to that discussion, also the hospitals who are not performing as well as the others, is great.’*

Furthermore, many participants were enthusiastic about the single-center short-cycle continuous quality improvement, amongst others through the use of electronic care pathways (subtheme b). For several of the participating teams, care pathways were built in the electronic information system. The care pathway enables easy extraction of systematically noted data and provides real-time outcomes and process indicators specific to a particular patient group. Several participants recognized the added value of such an electronic care pathway to enable shorter feedback and improvement cycles.

*‘So we have an EPIC- care pathway now, we just started. And with the use of the care path we can continuously see: “How did we perform last week?”. So this starts to look a lot like a continuous improvement cycle. Now, for the first time since EPIC was implemented, we can see how many patients we treat, how many new patients we see, and how often we operate. We now can control that.’*

### Availability of data

Data collection and analysis was considered time consuming and therefore mentioned as a hindering factor (subtheme a). Furthermore, access to data that was not directly related to an intervention (subtheme b) was also indicated as a hindering factor. According to participants, this type of data was not systematically registered and therefore relatively difficult to extract from electronic patient files:

*‘I think there is a lot of other data that is interesting but not necessarily easy to get. The first time out of bed would be a useful outcome for example. That would have been very interesting. But we haven’t registered it anywhere so it can’t be retrieved’.*

The presence of a (national) data registry was mentioned as a supporting factor for the availability of outcome data (subtheme c). An example that was mentioned many times is the Dutch Institute for Clinical Auditing (DICA), in which outcome data for a majority of oncological conditions is registered. Furthermore, participants considered the support of information technology (IT) an important factor (subtheme d) because members of the quality improvement team are most often not able to automatically extract data from the electronic patient file themselves. Therefore, many quality improvement teams are depending on the support and thus capacity of the hospital’s IT department.

## Discussion

The aim of this study was to explore the main factors that were experienced as hindering and/or supporting in the implementation of VBQI teams in hospital care. Overall, this study shows that the implementation of VBQI is rather complex. A successful implementation of VBQI takes time, requires solid change management and is dependent on many factors. We have identified nine main factors and several sub factors that were considered important in the implementation of VBQI teams. These nine main factors were divided into three overall domains: I. the organization of VBQI, II. the culture of VBQI, and III. the practice of VBQI. Domains found in the present study are consistent with domains that are described in many other innovation implementation studies [19]. Though, factors in the ‘practice’ domain are specific to value-based healthcare and have therefore not been described before.

Factors in the ‘organization’ and ‘practice’ domain of our study somewhat correspond with the first and second step of Porter and Lee’s value agenda, namely 1. ‘Organize into integrated practice units (IPUs)’, 2. ‘Measure outcomes and costs for every patient’. However, the present study provides more detailed practical handles for implementation. Factors that fall in the ‘culture’ domain of our study were not mentioned by Porter and Lee’s value agenda and are relatively new in the field of VBHC. Recently, a new strategic agenda for value transformation was proposed, adding four extra elements to the original agenda, including a specific notion of culture: ‘Set up value- based quality improvement’, ‘Integrate value in patient communication’, ‘Invest in a culture of value delivery (education)’, and ‘Build learning platforms for healthcare professionals’ [20].

Interestingly, the factors found in our study were equally relevant for all eight VBQI teams, regardless of their composition, patient characteristics or treatment options. We did however see that teams with a higher number of members experienced more trouble in the practical organization of the team meetings (planning and attendance of meetings). The two VBQI teams, hip fracture and sleep center, that started off with organizing in an IPU-like organization and thereafter started measuring and analyzing outcomes reported the same supporting and hindering factors as the other six VBQI teams did. The only difference was that they reported more positively about the current situation with regards to ‘organizing care around the patient’ and ‘shared workspace’. This seems to indicate that it is not necessary to implement the first and second step of Porter and Lee’s value agenda in that specific order.

### The organization of VBQI

Porter and Lee [3] recognize that the organization of care around the patient is an important step towards value-based healthcare. However, their advice to ‘organize into Integrated Practice Units (IPUs)’ does not provide detailed information on how to practically organize value-based care in a hospital setting. Present study indicates that a facilitating system (scheduled VBHC time, support staff) around the improvement team seems important. Not much research is done on this topic, but one of the few studies available is a Swedish study that acknowledged the benefits of an external consultancy firm in the organization support of VBQI teams [11].

In accordance with Porter and Lee’s value agenda, the present study highlights the importance of fully embedding VBHC in clinical practice. Sub factors such as ‘shared workspace’, ‘mandate of the improvement team’, ‘financial consequences of adopting VBHC concept’, ‘formal responsibility for quality of care’ etc., all suggest that there is need for more formal commitment to move the organization towards value-based healthcare. Correspondingly, a study looking into the organization of outcome-based quality improvement in Dutch heart centers found that the lack of governance - having no formalization of roles and responsibilities – was a barrier to the implementation of outcome-based quality improvement [21]. The same study also concluded that the implementation was unsuccessful when not embedded in the hospital strategy, policy documents, and planning and control [21].

Furthermore, present study emphasizes the need and opportunity for integrating VBHC with existing QI approaches such as the lean-philosophy. The combination of the lean-philosophy and VBHC has not yet been thoroughly researched before but the connectedness was already acknowledged in 2015 by a Swedish interview-study focusing on the understanding of value-based healthcare [10]. They stated that the lean-philosophy – just as VBHC – focuses on improving quality and efficiency by controlling costs (waste). A Dutch study looking into VBHC implementation also recognized that there is a need for a tool to connect outcomes and quality of care processes in order to organize a continuous improvement cycle [13]. The lean-philosophy could be that tool. The association between PROMs, SDM and VBHC has previously been researched, but not extensively. SDM has demonstrated to improve value for patients [22, 23] and outcome information has been indicated as the link between SDM and VBHC [24]. Moreover, a recent Dutch study that explored the association between PROMs, SDM and VBHC [25] showed that - at the patient level - PROMs were mainly used for monitoring and managing the medical condition. They also stated that more attention should be paid to the use of PROMs in SDM, especially during ‘choice talk’. In this part of SDM, patients’ values are considered and traded off in order to ultimately choose the treatment option that is of most value for the patient. At a macro level, PROMs and other patient-relevant outcomes can be compared between hospital organizations, to ultimately improve value for patients as a group.

### The culture of VBQI

The culture of VBQI - including main factors such as the ‘adoption of the VBHC concept’, ‘multidisciplinary engagement’ and ‘medical leadership’ – has not been thoroughly been researched in the VBHC field, nor was it explicitly mentioned in the value agenda of Porter and Lee [3]. The present study however shows that factors such as the adoption of the VBHC concept and multidisciplinary engagement are perceived as influential for the success of VBQI implementation in hospital care.

For the adoption of the VBHC concept, it was considered especially important that the reputation of the VBHC concept is positively perceived and that health professionals have enough knowledge about the VBHC theory. We saw that VBHC was especially positively perceived when people saw the added value (results) of VBHC. This would suggest that it can be stimulating to start with small improvements that produce quick results, so that health professionals get enthusiastic about the ability of VBQI to improve patient value. This finding is in agreement with the conclusion of Nilsson et al., (2017) who stated that their participants appreciated the VBHC concept because it focuses on increasing value for patients and gaining insight in health outcomes, as opposed to many other previous management interventions that focus mostly on measuring and controlling the costs. Furthermore, a study looking into health professionals’ perception of complex QI interventions, showed that health professionals are motivated to work towards a common QI goal when they have seen that the method works [26]. In our study we have indeed noticed that seeing the positive impact of an improvement initiative motivated the members to continue their work in the VBQIT. Another study focusing on organizational change in hospitals stated that the positive ánd negative expectations of health professionals in a hospital redevelopment depend on their level of understanding (of what change is to come) and on the level of resources and support they experience [27]. The present study indicates that improving the image of VBHC and the belief in the added value of the VBHC has potential to engage more health professionals in VBQI. A recent report from the Linnean Initiative in the Netherlands therefore called for the inclusion of VBHC in health professionals’ education [28]. The recently proposed new strategic agenda for value transformation also emphasized that education in VBHC is an important step [20].

In addition to the adoption of the VBHC concept, we found that multidisciplinary engagement was deemed valuable in the implementation of VBQI. There were two factors that were specific to VBQI: ‘outcome data that is relevant and adjustable for all team members’ and ‘patient involvement’. We found that in order for health professionals to stay engaged in the project, it is important that the data discussed in the meeting is relevant for all present team members. Therefore, it is recommended to organize several small meetings with only health professionals that have influence on outcome data that is discussed in that certain meeting. The other VBHC-specific sub factor that we found was patient involvement. As VBHC is about improving the value for the patient, it seems inevitable to include patients in the process of VBQI. A previous VBHC study stated that participants were surprised to see what patients considered valuable [11]. In our study, participants underscored the added value of patient participation, but also indicated that they struggled with finding the best way to involve patients in the process. To our knowledge, no research has yet been done on this topic. Therefore, we advise the VBHC research field to delve into the topic of patient participation in VBHC.

### The practice of VBQI

To support Porter and Lee’s [3] step ‘measure outcomes and costs for every patient’, the International Consortium for Health Outcomes Measurement (ICHOM) is developing international standard outcome sets for medical conditions. However, no practical advice is offered on whó should look at the outcome data, hów these health professionals should look at the outcome data, or how QI interventions should be selected and implemented. As a result, many hospitals have embraced the VBHC concept as an idea and have started QI-cycles, but many practical and methodological issues remain unanswered. The present study revealed the importance of factors as ‘goal setting and selecting quality improvement initiatives’, ‘long-cycle benchmarking and short-cycle feedback’ and the ‘availability of outcome data’.

Unanswered questions especially arose in the topic of setting clear goals for outcome improvement and selecting quality improvement initiatives. Interestingly, setting goals is a step that was not specifically mentioned in the Santeon improvement cycle [18] or in the value agenda [3]. There is no research available yet on how to set realistic improvement goals, on how many improvement goals should be set, or on how to set goals with a multidisciplinary team focused on patient value. Further research is necessary to improve the VBHC methodology. Moreover, until now, no ‘golden standard’ has been introduced as method for the selection of quality improvement initiatives based on outcome data. The lack of a clear methodology potentially complicates the process of setting improvement goals and ultimately selecting and implementing a QI initiative. In a previous study it was recognized that the process of VBQI was heavily modified over time due to the ambiguity and interpretable character of the VBHC concept [12]. Furthermore, van Veghel et al. [21] also identified the lack of a systematic approach to the identification and implementation of QI initiatives as a barrier. In response, Zipfel et al. [29] recently developed The Intervention Selection Toolbox for selecting interventions to improve patient-relevant outcomes in heart care. However, this toolbox is not yet validated for other medical conditions. Additionally, it is yet unclear how to increase value when outcomes fall within or above the national range of acceptance or when the outcome data is ‘average or above average’ compared to other hospitals. In the latter case, value improvement cannot be established through simply adopting best practices from other hospitals. The risk of benchmarking is that those who score above average can become less motivated to further improve themselves. It is therefore important to acknowledge that benchmarking outcomes is a learning process [30] and to develop an additional framework for value improvement when there is no clear improvement potential. Overall, the further development of the VBHC methodology requires more research as well as the integration of already existing knowledge from implementation science, organizational science and epidemiology into the VBHC research field. It is important for them to develop a different frame of reference in time to stimulate further improvement.

For the practical implementation of benchmarking, the present study observed that it was preferred to complement long-cycle benchmarking with short-cycle feedback. Short-cycle feedback through for example electronic care pathways ensures seeing quick results which leads to more motivation among participants to continue with the VBQI team [26]. Long-cycle quality improvement provides the ability to benchmark important long-term outcomes and identify improvement potential and best practices.

The availability of outcome data, or lack thereof, was experienced as an important factor in present study as well as in a previous VBHC studies [12, 21]. As technology advances we expect that electronic outcome data becomes more available in the future. Though, we noticed that especially structured outcome data, by means of electronic care pathways and (national) registries seems useful.

### Limitations

This study contains some limitations that need to be considered when interpreting the results of this study. First, open coding can possibly lead to interpretation bias. However, coding and analysis were independently checked by a second researcher. Furthermore, the open coding-method was thoughtfully chosen because of the innovative character of VBHC and the lack of an already existing framework to evaluate the implementation of VBHC. In the end, all three domains (organization/culture/practice) were comparable with domains described by general innovation implementation frameworks [19].

Second, due to the explorative design of this study and the use of open coding, we are unable to provide a point-by-point comparison between the factors found in this study and the steps in Porter and Lee’s value agenda. In sharing their experience, participants were not limited to the value agenda or any other framework. In future research, it could be interesting to question health professionals more specifically on their experience with each of the steps on Porter and Lee’s value agenda.

Third, two of the eight VBQI teams have a different starting point in their process of VBQI. These two teams started with organizing care around the patient, and thereafter started with measuring and analyzing outcome data. The other six participating teams started with measuring and analyzing outcome data. Although this could have influenced our results, we saw no apparent differences in the experience of VBQI between these two teams and the rest.

Fourth, interviews were performed by staff members of the hospital’s quality department who are also involved in the overall management and organization of VBHC and the lean-philosophy. This might have caused interpretation bias or socially desirable answers from participants, though we have tried to minimize the risks.

Fifth, this study is based on the experience of one hospital, which limits the generalizability of our results. Nevertheless, we have interviewed forty-three people from several improvement teams aiming to provide the most presentable results as possible. Further research in other hospitals is necessary to validate our results.

## Conclusion

The aim of this study was to explore the main factors that were experienced as hindering and/or supporting in the implementation of VBQI teams in hospital care. We have identified nine main factors, divided in three domains: I. the organization of VBQI, II. the culture of VBQI, and III. the practice of VBQI. Factors in the organizational and practical domain generally correspond to the first and second step of Porter and Lee’s value agenda but add practical handles on how to implement VBQI in hospital care. Surprisingly, the factors that we found in the cultural domain have not been pointed out in the value agenda and are relatively new in the field of VBHC [20]. Furthermore, findings of the present study suggest that it is not necessary to implement the first and second step of Porter and Lee’s agenda in that specific order. Further research should be done in order to validate our findings and further develop the VBHC methodology. Overall, the present study goes beyond the original value agenda and provides healthcare providers with more detailed knowledge on how to practically implement value-based quality improvement in a hospital care setting.

## Supplementary Information

**Additional file 1: Appendix A.** Interview guide.

**Additional file 2: Appendix B.** Composition of value-based quality improvement (VBQI) teams.

## Data Availability

The datasets used and/or analyzed during the current study are available from the corresponding author on reasonable request.
