# Supplementary material for: Five years’ experience with value-based quality improvement teams: the key factors to a successful implementation in hospital care
Source: BMC Health Serv Res. 2022 Oct 20;22:1271. doi: 10.1186/s12913-022-08563-5 (PMC9585830; doi:10.1186/s12913-022-08563-5)
Supplement: Supplementary file 1 — Additional file 1: Appendix A. Interview guide. [file 12913_2022_8563_MOESM1_ESM.docx]

**APPENDIX A: INTERVIEW GUIDE**

| **IMPLEMENTATION:**  HOW consistently is the intervention delivered, HOW will it be adapted and WHAT is the result? | To what extent are we able to successfully improve quality of care for condition X in a multidisciplinary and continuous way?  What is going well?  What is not going well or insufficiently well?  Which factors hinder continuous improvement of care for condition X?   - Inside/outside sphere of influence? - Who is responsible for improving this process?   How are decisions made about improvement initiatives that require multidisciplinary coordination?  How are improvement initiatives selected?   - How are they implemented? - Is this process going well?   To what extent does the hospital organization have the will and resources (adequate resources, knowledge and motivation) to implement disease-oriented care? |
| --- | --- |
| **EFFECTIVENESS:**  WHAT is the most important benefits you are trying to achieve and what is the likelihood of negative outcomes? | Which factors of the improvement team have contributed to improving the organizational or individual qualities?  To what extent has the deployment of the improvement team led to valuable results?  To what extent do the results of the multidisciplinary improvement team meet your expectations?  Have goals been formulated by the improvement team and are objectives achieved?   - If so, examples and what determines success? - If not, why not? - What is needed to improve this?   Is the improvement team effective in realizing improvement initiatives?  How does the improvement team receive feedback on its own performance?  To what extent does the deployment of improvement teams have adverse effects on the quality of care? |
| **ADOPTION:**  WHERE is the intervention applied and WHO applied it? | To what extent are all disciplines involved actually actively involved? (And are the right disciplines involved?)   - How important is this? - What does it take to increase engagement? - What could be a reason that colleagues participate less in an improvement team? - Which obstacles hinder participation? - To what extent do the various disciplines involved also feel responsible?   How are patients involved?   - How would you like to involve them? As part of the team?   To what extent are all healthcare professionals involved in the care of patients with condition X also involved in the improvement team? (And how?)  How are supportive care units involved (labs, pharmacy, etc.)?   - What is desirable in this? And what improvements are needed?   How are supporting units involved (KVV, Lean & Healthcare Logistics, I&I/BI, M&C, F&I)?   - What is desirable in this? - And what improvements are needed?   What mandate does the physician leading the improvement team need to be successful?   - How important is this physician's leadership? - Which roles are needed? In the team, or ad hoc   What do healthcare professionals need to be able to take control and responsibility for the quality of care for a patient group, i.e., related to a condition?   - Including nurse quality, PROMS, complaints, feedback radar, etc.   How does coordination take place with the partnerships and departments about substantive decisions?   - What is desirable in this? - And what improvements are needed? |
| **REACH:**  WHO is intended to benefit and who actually participates or is exposed to the intervention? | How many patients per year do we treat for this condition and how many of them have (probably) already noticed something concrete as a result of the improvement actions?  Does the improvement team have insight into the quality of care for all patients with condition X? Do the improvement actions also target all these patients?   - How can that number (%) be increased? |
| **MAINTENANCE:**  WHEN will the initiative become fully operational, how will it be sustained? | What are the biggest challenges during the implementation of improvement teams?  What does the hospital need to make improvement teams successful?  How can we structurally safeguard the working method of the improvement teams in the organization?   - What would you like to keep? - What would you like to change?   Are there any other relevant questions or topics that we haven't covered yet? |
