# Supplementary material for: Five years’ experience with value-based quality improvement teams: the key factors to a successful implementation in hospital care
Source: BMC Health Serv Res. 2022 Oct 20;22:1271. doi: 10.1186/s12913-022-08563-5 (PMC9585830; doi:10.1186/s12913-022-08563-5)
Supplement: Supplementary file 2 — Additional file 2: Appendix B. Composition of value-based quality improvement (VBQI) teams. [file 12913_2022_8563_MOESM2_ESM.docx]

APPENDIX B: Composition of value-based quality improvement (VBQI) teams

| **VBQI Team** | **Discipline** |
| --- | --- |
|  |  |
| Kidney failure | Medical leader, Internist / Nephrologist |
|  | Internist /nephrologist |
|  | Project leader |
|  | Head of Dialysis |
|  | Dialysis dietician |
|  | Dialysis nurse |
|  | Medical social worker |
|  | Researcher |
| Breast cancer | Medical Leader, Surgeon |
|  | Surgeon |
|  | Internist, oncologist |
|  | Plastic surgeon |
|  | Project leader |
|  | Head of Surgery |
|  | Nurse Specialist |
|  | Pathologist |
|  | Radiologist |
| Lung cancer | Medical leader, pulmonologist |
|  | Pulmonologist |
|  | Cardiothoracic surgeon |
|  | Project leader |
|  | Department head |
|  | 2x Nurse specialist |
|  | Pathologist |
|  | Hospital pharmacist |
| Sleep center | Medical leader, neurologist |
|  | 2x Pulmonologist |
|  | 2x ENT specialist |
|  | 2x Oral surgeon |
|  | Pediatric neurologist |
|  | Physician assistant |
|  | Dentist |
|  | 2x Department head |
|  | Team head |
|  | 2x Nurse |
|  | Care manager |
| Hip fracture | Medical leader, surgeon |
|  | Geriatric internist, CTO team |
|  | Emergency Medicine physician |
|  | Orthopedist |
|  | Head Orthopedic department |
|  | Head Emergency Medicine |
|  | Team head |
|  | 2x Physician assistant |
|  | 2x Nurse |
|  | Transfer nurse |
|  | Physician assistant |
|  | 2x Physiotherapist |
| Osteoarthritis | Medical leader, orthopedic surgeon |
|  | Project leader |
|  | 2x Department head |
|  | 2x Physiotherapist |
| Colon cancer | Medical leader, surgeon |
|  | GI specialist |
|  | 2x Nurse specialist |
|  | Nurse |
|  | Nurse (ostomy) |
|  | Project leader, researcher |
|  | Head nurse |
|  | Internist, oncologist |
|  | Pathologist |
| Prostate cancer | Medical leader, urologist, chef de clinique |
|  | Internist, oncologist |
|  | Project leader |
|  | Oncology nurse |
|  | Nuclear medicine specialist |
|  | Researcher |
